# Species richness and the dynamics of coral cover in Bangka Belitung Islands, Indonesia

**DOI:** 10.7717/peerj.14625

**Published:** 2023-02-24

**Authors:** Tri Aryono Hadi, Rizkie Satriya Utama, Tri Arfianti

**Affiliations:** 1 Research Center for Oceanography - National Research and Innovation Agency, North Jakarta, Jakarta, Indonesia; 2 Research Center for Biosystematics and Evolution - National Research and Innovation Agency, Cibinong, West Java, Indonesia

**Keywords:** Coral reefs, Species richness, Percent cover, Benthic communities, Dynamics of benthic covers, Bangka Belitung Islands

## Abstract

Pressures on the world’s tropical coral reefs that threaten their existence have been reported worldwide due to many stressors. Loss of coral cover and declines in coral richness are two of the most common changes often reported in coral reefs. However, a precise estimate of species richness and the coral cover dynamics for most Indonesian regions, particularly in the Bangka Belitung Islands, have been poorly documented. Annual monitoring data from 2015 to 2018 at 11 fixed sites in the Bangka Belitung Islands using the photo quadrat transect method identified 342 coral species from 63 genera. Of these, 231 species (>65%) were rare or uncommon, occurring in <40% of all sites. The species richness of hard corals was categorized as moderate compared to other studies in Indonesia, averaging 53 species across sites and years, and there was an increasing number of sites with high species richness. The percent cover of live and dead hard corals was greater than other benthic and substrate categories in all sites; revealing a live-dead hard corals pattern with dead coral cover averaged 12% higher than live hard coral across the years, but they did not show a significant difference (*P* > 0.05). There was a slightly increasing trend in hard coral cover in ten out of 11 sites in 2018, indicating the reefs are in a recovery process. The results support the need to identify recovering or stable areas despite apparent anthropogenic and natural variations recently. This vital information is essential for early detection and preparation for management strategies in the current context of climate change and for ensuring future coral reef survival.

## Introduction

Previous studies have reported various disturbances associated with climate change and anthropogenic factors to coral reefs worldwide, such as bleaching, pollution, overfishing, and destructive fishing practices (Jackson et al., 2001; Fox et al., 2003; McCulloch et al., 2003; Hughes et al., 2003). These disturbances might be unobserved (Mumby, 2017) but have been reported to influence coral reefs’ spatial and temporal dynamics (*e.g.*, Connell, 1978; Dollar & Tribble, 1993) and cause species diversity decline as well as community homogenization (Bak & Meesters, 1999; Osborne et al., 2011; Riegl & Purkis, 2012; Ortiz et al., 2018).

In Southeast Asia, more than 90% of coral reefs are at risk (Burke & Selig, 2002; Burke et al., 2011), including the risk from the El Ninõ phenomenon that occurs periodically and generates sea temperature rise. The rise initiates the breakdown of a mutual symbiotic relationship between corals and zooxanthellae, resulting in coral bleaching (Hoegh-Guldberg, 1999). One of the most recent and severe coral bleaching events was during the El Ninõ in 2016, which caused a dramatic decline in coral cover (*e.g.*, Ampou et al., 2017; Hughes et al., 2017; Ng et al., 2020). Another risk is associated with human activities, which have been intense in many big cities, making the urban reefs suffer from chronic stress (Heery et al., 2018).

Coral reef vulnerability has also been reported in Indonesia (Van Der Meij, Suharsono & Hoeksema, 2010). Reports on the status of the Indonesian major islands document environmental degradation due to pollution and land-based activities (Bird & Ongkosongo, 1980; Crawford et al., 2006; Farhan & Lim, 2012). The focus on economic-oriented activities due to the jurisdiction transfer from the central government to the local governments likewise has impacted the ecosystem in small islands (Farhan & Lim, 2010). Moreover, coral bleaching in 1998 and 2010 has been reported in Indonesian waters, which led to the mass death of corals in this region (Wouthuyzen, Abrar & Lorwens, 2018; Chaijaroen, 2019).

The Bangka Belitung Islands is a province comprising two major islands (Bangka and Belitung) and many small islands (*e.g.*, Mendanau, Nanduk, Ruk, Batu dinding, Sekutai) (Larasati, 2019). The province is located off Sumatra’s East Coast and is known for its coral reefs (Dermawan et al., 2014). In this case, the coral reefs are able to support the fishery sector, accounting for about 30% of the Bangka Belitung government’s total income (Sjafrie, 2009). However, the reefs have been reported to be under threat by natural and land-based activities, such as increasing sea surface temperature, sedimentation from mining activities, and the development of tourism (Putra et al., 2018; Syari & Nugraha, 2022). Moreover, an illegal, destructive fishing practice, especially using trawl, by local companies has also been reported in Bangka Belitung waters and its surroundings (Ivan, 2017).

Studies have highlighted the balance between reef-building and non-reef-building organisms and the importance of species richness and composition for the health and function of the ecosystem (Bellwood et al., 2004; Sweatman, Delean & Syms, 2011; Duffy, Godwin & Cardinale, 2017). However, as one of the archipelagic countries with over 17,000 small islands formed from carbonate and atolls with coral reefs (Bird & Ongkosongo, 1980), knowledge of species richness and the dynamics of coral cover in Indonesia is limited. To determine whether communities have experienced decreases in coral cover and diversity over time, this study recorded hard coral species richness and the dynamics of coral cover at 11 study sites with relatively good reef conditions in nine small islands in Belitung regency, Bangka Belitung province, Indonesia, from 2015 to 2018.

## Methods

### Sampling method

**Figure 1:**
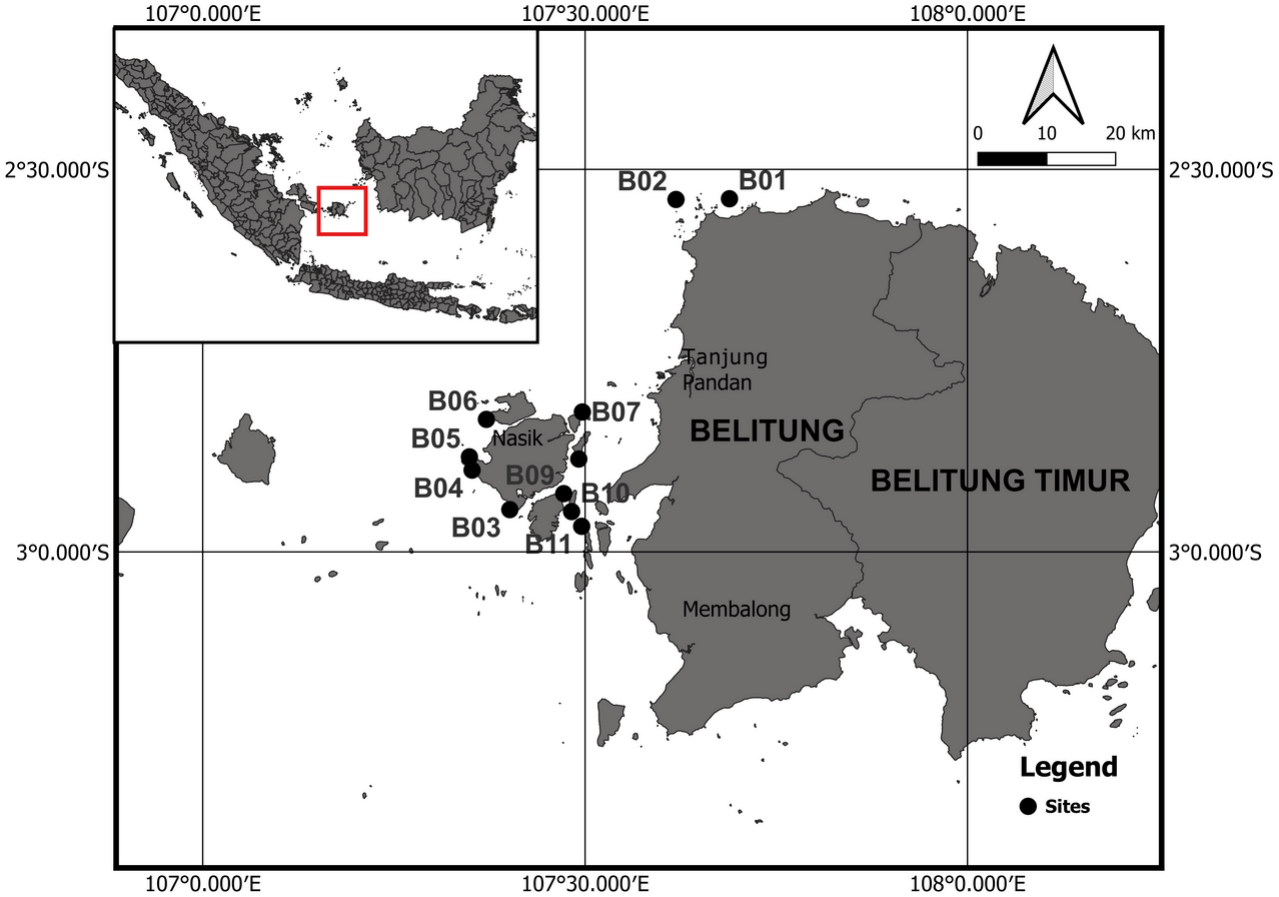
The map of study area in Bangka Belitung Islands.

The sampling was carried out at 11 fixed sites (Table 1) in nine small islands (Batu Malang Penyu, Lengkuas, Mendanau, Batu dinding, Sekutai, Sebongkok, Naduk, Sepindang, and Ruk) in Belitung regency, Bangka Belitung province (Fig. 1). The benthic composition was assessed annually from 2015 to 2018 using the photo quadrat transect method (Hill & Wilkinson, 2004) at approximately the same time each year (Table 1). All sites selected were located at outer slopes on exposed reefs that were assumed to have the highest percentage of the seabed covered by living hard coral, the highest populations of other biotas, and that were least affected by human activities. Therefore, the samplings were purposely biassed towards reefs in good condition. A 50-m transect line was placed parallel to the coastline on coral reefs in all sites at a relatively similar depth of 5 m–7 m (measured using the transect tape before photo data collection). Afterward, photos of a 44 × 58 cm frame were taken from meters 1 to 50 with an interval of 1 m (50 frame photos in total for one transect). To ensure the subsequent observations would take place on the same transect line or with no greater than 50 cm difference, permanent markers (50 cm iron sticks) were placed at the transect’s start, middle, and end. In addition, the coordinates of the starts of the transect lines were recorded with a portable Global Positioning System.

**Table 1:** Field study site locations, depth, and sampling date in Bangka Belitung Islands, Indonesia.

**Site**	**Depth (m)**	**Sampling date**				**Geographic position**	
		**2015**	**2016**	**2017**	**2018**	**Latitude**	**Longitude**
B01	5	25/9–4/10	10–20/10	1–10/12	29/8–9/9	−2.53838	107.68881
B02	5	25/9–4/10	10–20/10	1–10/12	29/8–9/9	−2.53934	107.61889
B03	5	25/9–4/10	10–20/10	1–10/12	29/8–9/9	−2.94477	107.40159
B04	5	25/9–4/10	10–20/10	1–10/12	29/8–9/9	−2.89315	107.35224
B05	5	25/9–4/10	10–20/10	1–10/12	29/8–9/9	−2.87579	107.34876
B06	5	25/9–4/10	10–20/10	1–10/12	29/8–9/9	−2.82678	107.37099
B07	5	25/9–4/10	10–20/10	1–10/12	29/8–9/9	−2.81713	107.49645
B08	5	25/9–4/10	10–20/10	1–10/12	29/8–9/9	−2.87862	107.49175
B09	5	25/9–4/10	10–20/10	1–10/12	29/8–9/9	−2.92386	107.47210
B10	5	25/9–4/10	10–20/10	1–10/12	29/8–9/9	−2.94733	107.48239
B11	5	25/9–4/10	10–20/10	1–10/12	29/8–9/9	−2.96661	107.49555

### Analyses

The frame photos were then analyzed using CPCe software to estimate benthic components’ percent covers (± SE) and substrates (Kohler & Gill, 2006). Thirty points were randomly deployed in each quadrant. If the points indicated live hard corals, then the corals were identified into species or genus levels following Veron & Stafford-Smith (2000). All identified live hard corals were standardized according to the Coral of The World website (Veron et al., 2022) to account for synonyms and taxonomic changes. All kinds of soft corals and fleshy macroalgae were difficult to distinguish in the images. Thus, they were all categorized into “soft corals” and “fleshy macroalgae”. CPCe only had one general sponge code that pooled all the species together. Thus, even though some sites had more than one type of sponge present, they were all categorized into “sponge”. Coralline algae, *Halimeda*, zoanthid, and other fauna were pooled into “other biota”. Only live hard corals were identified to the species level, and only live hard coral species were included in calculations of species richness for each site. Recent dead hard coral and dead hard coral covered by turf algae were identified as dead coral. Hard coral cover died recently at less than 2% data across all stations, and we missed the peak heat stress events; hence we failed to record the impact on corals. The substrate was classified into four categories, including sand, broken dead coral (rubble), silt, and natural rock. The status of the surveyed coral reefs is defined by the category of hard coral cover *i.e.,* low (>10%–10%), moderate (>10%–30%), high (>30%–50%), very high (>50%–75%), and extremely high >75%–100% following classification of the Australian Institute of Marine Science (AIMS , 2021).

Percentage coral cover data were prepared using arcsine transformation prior to repeated measures ANOVA analyses (Sokal, 1995; Zar, 1999). Repeated measures ANOVA was used due to repeated sampling of the same transects. Sites were treated as statistical replicates, time as the repeated factor, and cover data from photo quadrats were averaged by the site to describe community structure with 11 replicates per year. Percent cover of each benthic category between sites and monitoring time is displayed in bar charts, while changes over time in the percentage cover of hard and soft corals are displayed using line graphs, showing means ±SE for untransformed values.

In terms of variation in coral composition, this study used an ordination analysis at the genera level, instead of the species level, to retain the robustness of the result while maintaining sensitivity to environmental changes (Somerfield & Clarke, 1995). The data were fourth-root transformed to improve the spread of the data and then standardized to rescale the data to have a mean of 0 and a standard deviation of 1. Afterward, the data were analyzed by non-metric multidimensional scaling (nMDS) analysis on a Bray Curtis dissimilarity matrix using Primer 7 software (Clarke et al., 2014).

## Results

**Figure 2:**
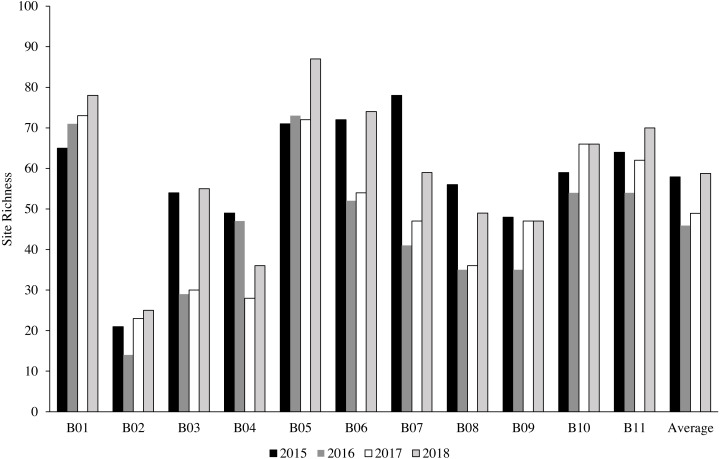
The percentage of coral species richness at each station throughout the years.

**Figure 3:**
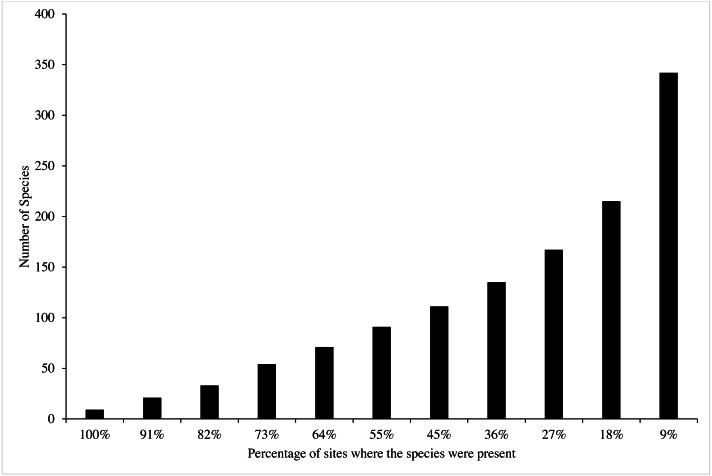
The percentage of coral species found in Bangka Belitung Islands.

A total of 342 species from 63 genera were identified at 11 sites from 2015 to 2018 (Table 2). The most common genera were *Acropora* with 45 recorded species, *Montipora* with 32 species and *Porites* with 21 species (Table 3). Bangka Belitung Islands’ richness averaged 46 overall sites and years (range 8–88; Table 4). B02 had the lowest richness, and B05 had the highest richness among all of the sites across the years (Fig. 2, Table 4). In 2015, 70% of sites had high species richness (>50 species per site), which decreased to 45% in 2016 (Fig. 3). In 2017, all sites had low species richness (<35 species per site), but recovered in 2018, with 45% of sites having >65 species per site, 35% of sites having >45 species per site, and the rest of the sites having ≥ 25 species per site (Fig. 3).

**Table 2:** Checklist of live hard coral species in Bangka Belitung Waters, sorted according to the largest cover over sites and year, and the IUCN status of the species.

Species	Number of sites species occurred	Occurrence percentage of site	Average cover over site and year	IUCN status
*Galaxea astreata*	8	73%	9.58%	VU
*Echinopora mammiformis*	6	55%	5.80%	NT
*Porites lutea*	11	100%	3.44%	LC
*Goniopora pandoraensis*	1	9%	3.10%	LC
*Montipora aequituberculata*	2	18%	2.53%	LC
*Goniopora planulata*	2	18%	2.53%	VU
*Acropora spicifera*	1	9%	2.50%	VU
*Porites lobata*	10	91%	2.34%	NT
*Leptoseris yabei*	1	9%	2.27%	VU
*Porites rus*	11	100%	2.08%	LC
*Goniopora eclipsensis*	1	9%	2.03%	LC
*Porites horizontalata*	7	64%	1.93%	VU
*Merulina scabricula*	11	100%	1.84%	LC
*Montipora foliosa*	8	73%	1.65%	NT
*Diploastrea heliopora*	10	91%	1.43%	NT
*Oxypora* sp.	1	9%	1.43%	
*Goniopora tenuidens*	1	9%	1.30%	LC
*Echinopora lamellosa*	9	82%	1.27%	LC
*Mycedium robokaki*	8	73%	1.23%	LC
*Montipora crassituberculata*	7	64%	1.20%	VU
*Porites monticulosa*	8	73%	1.18%	LC
*Pachyseris rugosa*	7	64%	1.18%	VU
*Hydnophora* sp.	1	9%	1.15%	
*Acropora hyacinthus*	5	45%	1.13%	NT
*Pachyseris speciosa*	9	82%	1.12%	LC
*Merulina ampliata*	10	91%	1.01%	LC
*Lobophyllia hemprichii*	10	91%	1.01%	LC
*Mycedium elephantotus*	8	73%	0.96%	LC
*Porites cylindrica*	11	100%	0.94%	NT
*Podabacia motuporensis*	1	9%	0.93%	NT
*Pectinia alcicornis*	8	73%	0.92%	VU
*Porites lichen*	6	55%	0.83%	LC
*Porites nigrescens*	8	73%	0.81%	VU
*Platygyra daedalea*	1	9%	0.78%	LC
*Echinopora pacificus*	10	91%	0.76%	NT
*Echinophyllia* sp.	4	36%	0.75%	
*Oxypora glabra*	9	82%	0.75%	LC
*Mycedium mancaoi*	4	36%	0.73%	LC
*Stylophora subseriata*	5	45%	0.72%	LC
*Montipora friabilis*	1	9%	0.71%	VU
*Fungia danai*	10	91%	0.71%	LC
*Turbinaria reniformis*	9	82%	0.70%	VU
*Fungia concinna*	11	100%	0.69%	LC
*Lobophyllia corymbosa*	8	73%	0.68%	LC
*Acropora abrotanoides*	5	45%	0.67%	LC
*Porites densa*	1	9%	0.67%	NT
*Hydnophora microconos*	1	9%	0.64%	NT
*Oulophyllia crispa*	4	36%	0.63%	NT
*Pavona decussata*	8	73%	0.63%	VU
*Montipora efflorescens*	6	55%	0.61%	NT
*Oxypora lacera*	8	73%	0.59%	LC
*Acropora cytherea*	5	45%	0.59%	LC
*Pectinia paeonia*	9	82%	0.58%	NT
*Acanthastrea subechinata*	2	18%	0.57%	NT
*Acropora digitifera*	2	18%	0.57%	NT
*Porites annae*	3	27%	0.57%	NT
*Porites latistella*	2	18%	0.56%	LC
*Galaxea cryptoramosa*	3	27%	0.55%	VU
*Porites solida*	4	36%	0.53%	LC
*Pectinia ayleni*	1	9%	0.53%	NT
*Isopora brueggemanni*	3	27%	0.53%	VU
*Galaxea fascicularis*	11	100%	0.52%	NT
*Acropora millepora*	7	64%	0.51%	NT
*Platygyra ryukyuensis*	8	73%	0.50%	NT
*Montastrea curta*	2	18%	0.50%	LC
*Goniastrea pectinata*	10	91%	0.49%	LC
*Porites vaughani*	2	18%	0.49%	LC
*Caulastrea curvata*	1	9%	0.49%	VU
*Porites australiensis*	1	9%	0.48%	LC
*Astreopora randalli*	1	9%	0.47%	LC
*Pavona frondifera*	8	73%	0.45%	LC
*Pavona explanulata*	4	36%	0.45%	LC
*Echinopora horrida*	4	36%	0.44%	NT
*Turbinaria peltata*	1	9%	0.43%	VU
*Montipora* sp.	11	100%	0.42%	
*Pectinia lactuca*	9	82%	0.42%	VU
*Cyphastrea* sp.	3	27%	0.42%	
*Pavona danai*	1	9%	0.42%	VU
*Platygyra sinensis*	6	55%	0.41%	LC
*Goniopora lobata*	7	64%	0.41%	NT
*Montipora turtlensis*	2	18%	0.40%	VU
*Acanthastrea rotundoflora*	1	9%	0.40%	NT
*Acropora paniculata*	1	9%	0.40%	VU
*Favites pentagona*	2	18%	0.40%	LC
*Astreopora suggesta*	4	36%	0.40%	LC
*Caulastrea furcata*	5	45%	0.39%	LC
*Favites russelli*	7	64%	0.38%	NT
*Astreopora myriophthalma*	7	64%	0.36%	LC
*Turbinaria mesenterina*	3	27%	0.36%	VU
*Fungia scabra*	9	82%	0.35%	LC
*Echinophyllia aspera*	7	64%	0.35%	LC
*Montipora monasteriata*	9	82%	0.34%	LC
*Porites deformis*	1	9%	0.33%	NT
*Echinophyllia echinoporoides*	1	9%	0.33%	LC
*Goniastrea minuta*	5	45%	0.33%	NT
*Montipora peltiformis*	3	27%	0.33%	NT
*Fungia repanda*	7	64%	0.33%	LC
*Oxypora crassispinosa*	6	55%	0.33%	LC
*Goniastrea retiformis*	6	55%	0.33%	LC
*Acropora insignis*	8	73%	0.33%	DD
*Favites complanata*	10	91%	0.32%	NT
*Acropora latistella*	3	27%	0.32%	LC
*Fungia fungites*	10	91%	0.32%	NT
*Platygyra pini*	6	55%	0.32%	LC
*Sandalolitha* sp.	2	18%	0.31%	
*Goniastrea favulus*	10	91%	0.30%	NT
*Favites halicora*	7	64%	0.30%	NT
*Montipora corbettensis*	2	18%	0.30%	VU
*Acropora caroliniana*	2	18%	0.30%	VU
*Acropora divaricata*	5	45%	0.30%	NT
*Acropora selago*	2	18%	0.30%	NT
*Acropora tenuis*	7	64%	0.30%	NT
*Favia amicorum*	2	18%	0.30%	LC
*Favites micropentagona*	4	36%	0.30%	NT
*Acropora microphthalma*	1	9%	0.30%	LC
*Pavona cactus*	7	64%	0.29%	VU
*Symphyllia recta*	6	55%	0.28%	LC
*Physogyra lichtensteini*	11	100%	0.28%	VU
*Pocillopora damicornis*	6	55%	0.27%	LC
*Favia speciosa*	10	91%	0.27%	LC
*Acropora samoensis*	5	45%	0.27%	LC
*Stylocoeniella cocosensis*	1	9%	0.27%	VU
*Symphyllia agaricia*	8	73%	0.27%	LC
*Acropora abrolhosensis*	1	9%	0.27%	VU
*Acropora desalwii*	1	9%	0.27%	VU
*Acropora granulosa*	1	9%	0.27%	NT
*Acropora plana*	1	9%	0.27%	DD
*Euphyllia yaeyamaensis*	1	9%	0.27%	NT
*Goniastrea australensis*	3	27%	0.27%	LC
*Leptoria irregularis*	2	18%	0.27%	VU
*Millepora platyphylla*	1	9%	0.27%	LC
*Montipora hodgsoni*	1	9%	0.27%	VU
*Phymastrea colemani*	3	27%	0.27%	NT
*Platygyra contorta*	3	27%	0.27%	LC
*Montipora cebuensis*	1	9%	0.27%	VU
*Psammocora digitata*	2	18%	0.27%	NT
*Cyphastrea serailia*	8	73%	0.26%	LC
*Turbinaria stellulata*	6	55%	0.25%	VU
*Lobophyllia robusta*	3	27%	0.24%	LC
*Acanthastrea echinata*	3	27%	0.23%	LC
*Acropora florida*	2	18%	0.23%	NT
*Acropora pulchra*	2	18%	0.23%	LC
*Montipora delicatula*	3	27%	0.23%	VU
*Montipora millepora*	3	27%	0.23%	LC
*Porites rugosa*	1	9%	0.23%	VU
*Heliopora coerulea*	5	45%	0.23%	VU
*Platygyra verweyi*	5	45%	0.23%	NT
*Cyphastrea ocellina*	2	18%	0.22%	VU
*Symphyllia radians*	7	64%	0.22%	LC
*Ctenactis echinata*	10	91%	0.22%	LC
*Lobophyllia hataii*	1	9%	0.22%	LC
*Goniopora minor*	9	82%	0.22%	NT
*Montipora informis*	4	36%	0.21%	LC
*Goniastrea palauensis*	6	55%	0.21%	NT
*Leptastrea purpurea*	9	82%	0.21%	LC
*Acropora loripes*	3	27%	0.20%	NT
*Acropora aspera*	1	9%	0.20%	VU
*Acropora carduus*	1	9%	0.20%	NT
*Acropora clathrata*	1	9%	0.20%	LC
*Acropora striata*	1	9%	0.20%	VU
*Acropora subulata*	1	9%	0.20%	LC
*Astreopora expansa*	1	9%	0.20%	NT
*Ctenactis crassa*	6	55%	0.20%	NT
*Leptastrea bewickensis*	1	9%	0.20%	NT
*Millepora exaesa*	2	18%	0.20%	LC
*Montipora capricornis*	2	18%	0.20%	VU
*Montipora malampaya*	1	9%	0.20%	VU
*Montipora tuberculosa*	4	36%	0.20%	LC
*Turbinaria irregularis*	1	9%	0.20%	LC
*Acropora cerealis*	3	27%	0.20%	LC
*Psammocora contigua*	4	36%	0.20%	NT
*Paramontastraea salebrosa*	1	9%	0.20%	VU
*Porites* sp.	11	100%	0.19%	
*Fungia fralinae*	5	45%	0.19%	LC
*Platygyra lamellina*	8	73%	0.19%	NT
*Goniastrea aspera*	3	27%	0.19%	LC
*Favia danae*	4	36%	0.19%	LC
*Heliofungia actiniformis*	5	45%	0.18%	VU
*Acanthastrea hemprichii*	3	27%	0.18%	VU
*Astreopora ocellata*	3	27%	0.18%	LC
*Favia lizardensis*	4	36%	0.18%	NT
*Favites abdita*	8	73%	0.17%	NT
*Favia veroni*	6	55%	0.17%	NT
*Goniastrea edwardsi*	7	64%	0.17%	LC
*Favia stelligera*	1	9%	0.17%	NT
*Porites eridani*	1	9%	0.17%	EN
*Symphyllia valenciennesii*	2	18%	0.17%	LC
*Turbinaria* sp.	2	18%	0.17%	VU
*Favites paraflexuosa*	6	55%	0.17%	NT
*Fungia paumotensis*	8	73%	0.16%	LC
*Acropora* sp.	7	64%	0.16%	
*Goniopora columna*	9	82%	0.16%	NT
*Fungia granulosa*	2	18%	0.16%	LC
*Favites flexuosa*	6	55%	0.16%	NT
*Euphyllia ancora*	5	45%	0.16%	VU
*Goniopora palmensis*	2	18%	0.16%	LC
*Plesiastrea versipora*	1	9%	0.16%	LC
*Pavona venosa*	5	45%	0.15%	VU
*Pectinia teres*	2	18%	0.15%	NT
*Astreopora gracilis*	4	36%	0.15%	LC
*Euphyllia glabrescens*	6	55%	0.14%	NT
*Favites vasta*	5	45%	0.14%	NT
*Favia matthaii*	3	27%	0.14%	NT
*Montipora caliculata*	4	36%	0.14%	LC
*Podabacia crustacea*	6	55%	0.14%	LC
*Cyphastrea microphthalma*	8	73%	0.14%	LC
*Fungia* sp.	9	82%	0.14%	
*Parascolymia vitiensis*	1	9%	0.13%	LC
*Acanthastrea hillae*	3	27%	0.13%	NT
*Acanthastrea ishigakiensis*	1	9%	0.13%	VU
*Acropora aculeus*	1	9%	0.13%	VU
*Acropora sarmentosa*	1	9%	0.13%	LC
*Acropora valida*	1	9%	0.13%	LC
*Acropora verweyi*	1	9%	0.13%	VU
*Coscinaraea columna*	1	9%	0.13%	LC
*Echinophyllia patula*	1	9%	0.13%	LC
*Euphyllia divisa*	2	18%	0.13%	NT
*Favia maritima*	7	64%	0.13%	NT
*Fungia molluccensis*	1	9%	0.13%	LC
*Halomitra pileus*	1	9%	0.13%	LC
*Leptastrea transversa*	3	27%	0.13%	LC
*Leptoria phrygia*	2	18%	0.13%	NT
*Leptoseris* sp.	2	18%	0.13%	
*Merulina* sp.	1	9%	0.13%	
*Montipora effusa*	1	9%	0.13%	NT
*Montipora florida*	1	9%	0.13%	VU
*Montipora hispida*	3	27%	0.13%	LC
*Montipora nodosa*	2	18%	0.13%	NT
*Montipora spumosa*	1	9%	0.13%	LC
*Pavona clavus*	1	9%	0.13%	VU
*Phymastrea magnistellata*	1	9%	0.13%	NT
*Platygyra yaeyamaensis*	1	9%	0.13%	VU
*Porites attenuata*	1	9%	0.13%	NT
*Porites stephensoni*	4	36%	0.13%	NT
*Favia* sp.	8	73%	0.13%	
*Fungia klunzingeri*	6	55%	0.13%	NE
*Plerogyra sinuosa*	8	73%	0.13%	NT
*Favia rotundata*	5	45%	0.12%	NT
*Montipora turgescens*	4	36%	0.12%	LC
*Montipora venosa*	4	36%	0.12%	NT
*Favia pallida*	4	36%	0.12%	LC
*Pavona varians*	7	64%	0.12%	LC
*Coscinaraea exesa*	2	18%	0.12%	LC
*Goniopora* sp.	4	36%	0.12%	
*Lithophyllon undulatum*	3	27%	0.12%	NT
*Psammocora profundacella*	5	45%	0.12%	LC
*Favites chinensis*	5	45%	0.12%	NT
*Favia favus*	5	45%	0.11%	LC
*Leptastrea pruinosa*	2	18%	0.11%	LC
*Millepora* sp.	2	18%	0.11%	
*Platygyra* sp.	2	18%	0.11%	
*Herpolitha limax*	6	55%	0.11%	LC
*Cyphastrea japonica*	3	27%	0.11%	LC
*Alveopora spongiosa*	4	36%	0.10%	NT
*Echinophyllia orpheensis*	5	45%	0.10%	LC
*Acanthastrea* sp.	3	27%	0.10%	
*Montastrea* sp.	2	18%	0.10%	
*Acropora humilis*	1	9%	0.10%	NT
*Acropora indonesia*	1	9%	0.10%	VU
*Coscinaraea monile*	2	18%	0.10%	LC
*Ctenactis* sp.	2	18%	0.10%	
*Cyphastrea chalcidicum*	2	18%	0.10%	LC
*Echinopora ashmorensis*	1	9%	0.10%	VU
*Echinopora gemmacea*	1	9%	0.10%	LC
*Hydnophora rigida*	1	9%	0.10%	LC
*Montipora hoffmeisteri*	2	18%	0.10%	LC
*Montipora orientalis*	1	9%	0.10%	VU
*Pachyseris* sp.	2	18%	0.10%	
*Pocillopora verrucosa*	1	9%	0.10%	LC
*Echinopora* sp.	3	27%	0.09%	
*Platygyra acuta*	3	27%	0.09%	NT
*Favites* sp.	6	55%	0.09%	
*Sandalolitha robusta*	3	27%	0.09%	LC
*Favites acuticollis*	3	27%	0.08%	NT
*Gardineroseris planulata*	4	36%	0.08%	LC
*Oulophyllia bennettae*	3	27%	0.08%	NT
*Acanthastrea bowerbanki*	1	9%	0.07%	VU
*Acanthastrea faviaformis*	1	9%	0.07%	VU
*Acropora austera*	1	9%	0.07%	NT
*Acropora bifurcata*	1	9%	0.07%	DD
*Acropora fastigata*	1	9%	0.07%	DD
*Acropora gemmifera*	1	9%	0.07%	LC
*Acropora horrida*	1	9%	0.07%	VU
*Acropora kimbeensis*	2	18%	0.07%	VU
*Acropora muricata*	1	9%	0.07%	NT
*Acropora nasuta*	2	18%	0.07%	NT
*Acropora secale*	1	9%	0.07%	NT
*Acropora speciosa*	1	9%	0.07%	VU
*Alveopora allingi*	1	9%	0.07%	VU
*Alveopora minuta*	1	9%	0.07%	EN
*Coscinaraea crassa*	2	18%	0.07%	NT
*Cycloseris costulata*	1	9%	0.07%	LC
*Echinophyllia echinata*	1	9%	0.07%	LC
*Favia helianthoides*	1	9%	0.07%	NT
*Favia maxima*	2	18%	0.07%	NT
*Favia rotumana*	1	9%	0.07%	LC
*Favites bestae*	1	9%	0.07%	NT
*Galaxea longisepta*	1	9%	0.07%	NT
*Goniastrea* sp.	5	45%	0.07%	
*Goniopora djiboutiensis*	1	9%	0.07%	LC
*Goniopora pendulus*	1	9%	0.07%	LC
*Halomitra clavator*	1	9%	0.07%	VU
*Hydnophora exesa*	2	18%	0.07%	NT
*Hydnophora grandis*	1	9%	0.07%	LC
*Hydnophora pilosa*	1	9%	0.07%	LC
*Isopora palifera*	1	9%	0.07%	NT
*Leptastrea* sp.	1	9%	0.07%	
*Leptoseris explanata*	1	9%	0.07%	LC
*Leptoseris scabra*	1	9%	0.07%	LC
*Lithophyllon* sp.	1	9%	0.07%	
*Lobophyllia pachysepta*	2	18%	0.07%	NT
*Micromussa amakusensis*	1	9%	0.07%	NT
*Micromussa* sp.	1	9%	0.07%	
*Millepora dichotoma*	1	9%	0.07%	LC
*Montipora cocosensis*	2	18%	0.07%	VU
*Montipora floweri*	1	9%	0.07%	LC
*Montipora palawanensis*	1	9%	0.07%	NT
*Montipora stellata*	1	9%	0.07%	LC
*Moseleya latistellata*	1	9%	0.07%	VU
*Mycedium* sp.	1	9%	0.07%	
*Parascolymia australis*	1	9%	0.07%	LC
*Pavona minuta*	1	9%	0.07%	NT
*Pavona* sp.	4	36%	0.07%	
*Pectinia* sp.	3	27%	0.07%	
*Phymastrea valenciennesi*	2	18%	0.07%	NT
*Platygyra carnosus*	1	9%	0.07%	NT
*Pocillopora danae*	1	9%	0.07%	VU
*Pocillopora* sp.	1	9%	0.07%	
*Porites negrosensis*	1	9%	0.07%	NT
*Psammocora explanulata*	1	9%	0.07%	LC
*Psammocora* sp.	1	9%	0.07%	
*Pseudosiderastrea tayami*	1	9%	0.07%	NT
*Sandalolitha dentata*	1	9%	0.07%	LC
*Seriatopora stellata*	1	9%	0.07%	NT
*Siderastrea savignyana*	1	9%	0.07%	LC
*Stylophora pistillata*	1	9%	0.07%	NT
*Symphyllia hassi*	1	9%	0.07%	LC
*Symphyllia* sp.	1	9%	0.07%	
*Tubastrea* sp.	1	9%	0.07%	
*Turbinaria frondens*	1	9%	0.07%	LC
*Turbinaria radicalis*	1	9%	0.07%	VU

**Table 3:** List of live hard coral genera found in this study and their number of species.

**Live hard coral**	**Number of species**
Acropora	45
Montipora	32
Porites	21
Favia	15
Favites	13
Fungia	11
Goniopora	11
Platygyra	11
Pavona	10
Acanthastrea	9
Goniastrea	9
Turbinaria	8
Echinopora	7
Astreopora	6
Cyphastrea	6
Echinophyllia	6
Hydnophora	6
Pectinia	6
Psammocora	6
Symphyllia	6
Galaxea	5
Leptastrea	5
Lobophyllia	5
Coscinaraea	4
Euphyllia	4
Leptoseris	4
Millepora	4
Mycedium	4
Oxypora	4
Pocillopora	4
Alveopora	3
Ctenactis	3
Cycloseris	3
Merulina	3
Pachyseris	3
Phymastrea	3
Sandalolitha	3
Caulastrea	2
Halomitra	2
Isopora	2
Leptoria	2
Lithophyllon	2
Micromussa	2
Montastrea	2
Oulophyllia	2
Parascolymia	2
Podabacia	2
Stylophora	2
Diploastrea	1
Gardineroseris	1
Heliofungia	1
Heliopora	1
Herpolitha	1
Moseleya	1
Paramontastraea	1
Physogyra	1
Plerogyra	1
Plesiastrea	1
Pseudosiderastrea	1
Seriatopora	1
Siderastrea	1
Stylocoeniella	1
Tubastrea	1

**Table 4:** The species richness of hard corals for each site.

**Site**	**2015**		**2016**		**2017**		**2018**		**Mean**		**SD**		**Range**	
	**SpR**	**H’**	**SpR**	**H’**	**SpR**	**H’**	**SpR**	**H’**	**SpR**	**H’**	**SpR**	**H’**	**SpR**	**H’**
B01	65	2.11	71	2.81	73	2.92	78	2.71	72	2.64	5	0.36	65–78	2.11–2.92
B02	21	0.85	14	0.87	23	1.05	25	1.11	21	0.97	5	0.13	14–25	0.85–1.11
B03	54	2.86	29	2.37	30	2.44	55	3.11	42	2.7	14	0.35	29–55	2.37–3.11
B04	49	2.51	47	2.47	28	2.47	36	2.41	40	2.47	10	0.04	28–49	2.41–2.51
B05	71	3.45	73	3.52	72	3.51	87	3.69	76	3.55	8	0.1	71–87	3.45–3.69
B06	72	3.35	52	2.63	54	2.85	74	3.02	63	2.96	12	0.3	52–74	2.63–3.35
B07	78	3.6	41	2.68	47	3.03	59	3.08	56	3.1	16	0.38	41–78	2.68–3.6
B08	56	3.35	35	2.92	36	3.01	49	3.25	44	3.13	10	0.2	35–56	2.92–3.35
B09	48	2.61	35	2.37	47	2.64	47	2.39	44	2.5	6	0.14	35–48	2.37–2.64
B10	60	3.3	54	3.18	66	3.62	66	3.52	62	3.4	6	0.2	54–66	3.18–3.62
B11	64	3.32	54	3.36	62	3.42	70	3.45	63	3.39	7	0.06	54–70	3.32–3.45
**Mean**	58	2.85	46	2.65	49	2.81	59	2.89	53	2.8				
**SD**	16	0.81	18	0.71	18	0.71	19	0.73						
**Range**	21–78	0.85–3.6	14–73	0.87–3.52	23–73	1.05–3.62	25–87	1.11–3.69						

**Notes.**

* noted: SpR (species richness), H’(Shannon-Weiner Index).

Most of the 342 recorded species were rare or uncommon (231 species), occurring in only a small percentage (<40%) of the sites surveyed (Fig. 3). About 50% of the species were recorded in less than 20% of the sites, and 33 species were recorded in >80%. Only nine species were documented in all sites (Fig. 3, Table 2). Of these, three species were from the genus *Porites*, *i.e., Porites lutea*, *Porites cylindrica*, *Porites* sp. According to the IUCN Red List, most of the hard coral species identified in this study were classified as Least Concern (43.69%), Near Threatened (32.69%), and Vulnerable (21.36%). Two species, *Porites eridani* and *Alveopora minuta* were identified as Endangered. The remaining species were Not Evaluated (0.32%) and were Data Deficient (1.29%) (Table 2).

**Figure 4:**
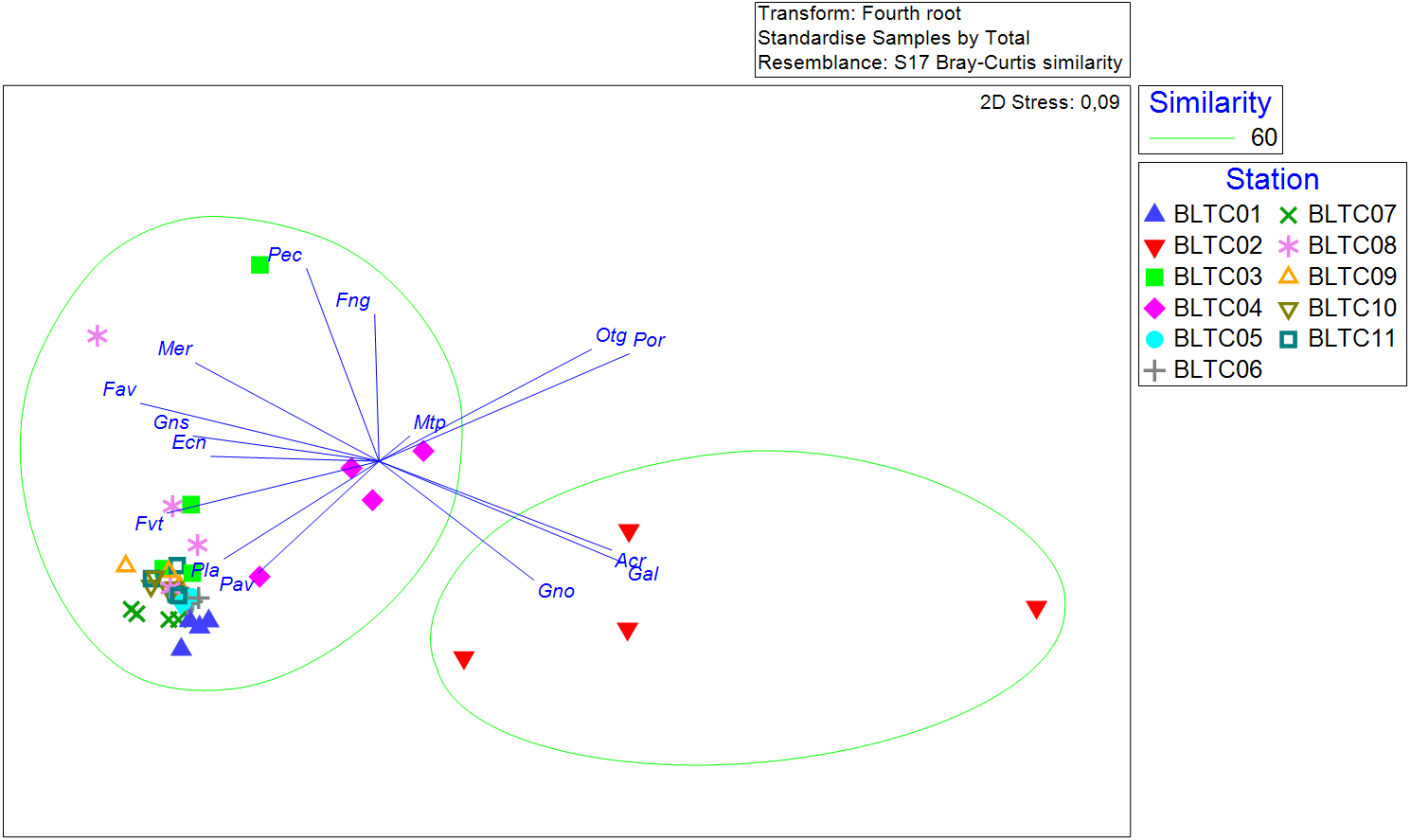
The non-metric multidimensional scaling of coral genera’s percent covers at each site across the years. Acr: *Acropora*, Ecn: *Echinopora*, Fav: *Favia*, Fng: *Fungia*, Fvt: *Favites*, Gal: *Galaxea*, Gno: *Goniopora,* Gns: *Goniastrea,* Mer: *Merulina*, Mtp: *Montipora*, Otg: other genera, Pav: *Pavona*, Pec: *Pectinia,* Pla: *Platygyra*, Por: *Porites*.

In terms of coral composition, especially at the genera level, live hard corals in Bangka Belitung islands are divided into two different clusters. The first cluster is dominated by *Acropora*, *Galaxea* and *Goniopora* occupying B02, whereas the other cluster is more diverse, featuring plenty of genera, and occurs in the rest of the sites (Fig. 4). Furthermore, it shows that the composition does not vary among the years; given that the year samples from the same sites are relatively close to each other and gathered in the same cluster. Hence, the coral composition varies spatially but not temporally.

**Figure 5:**
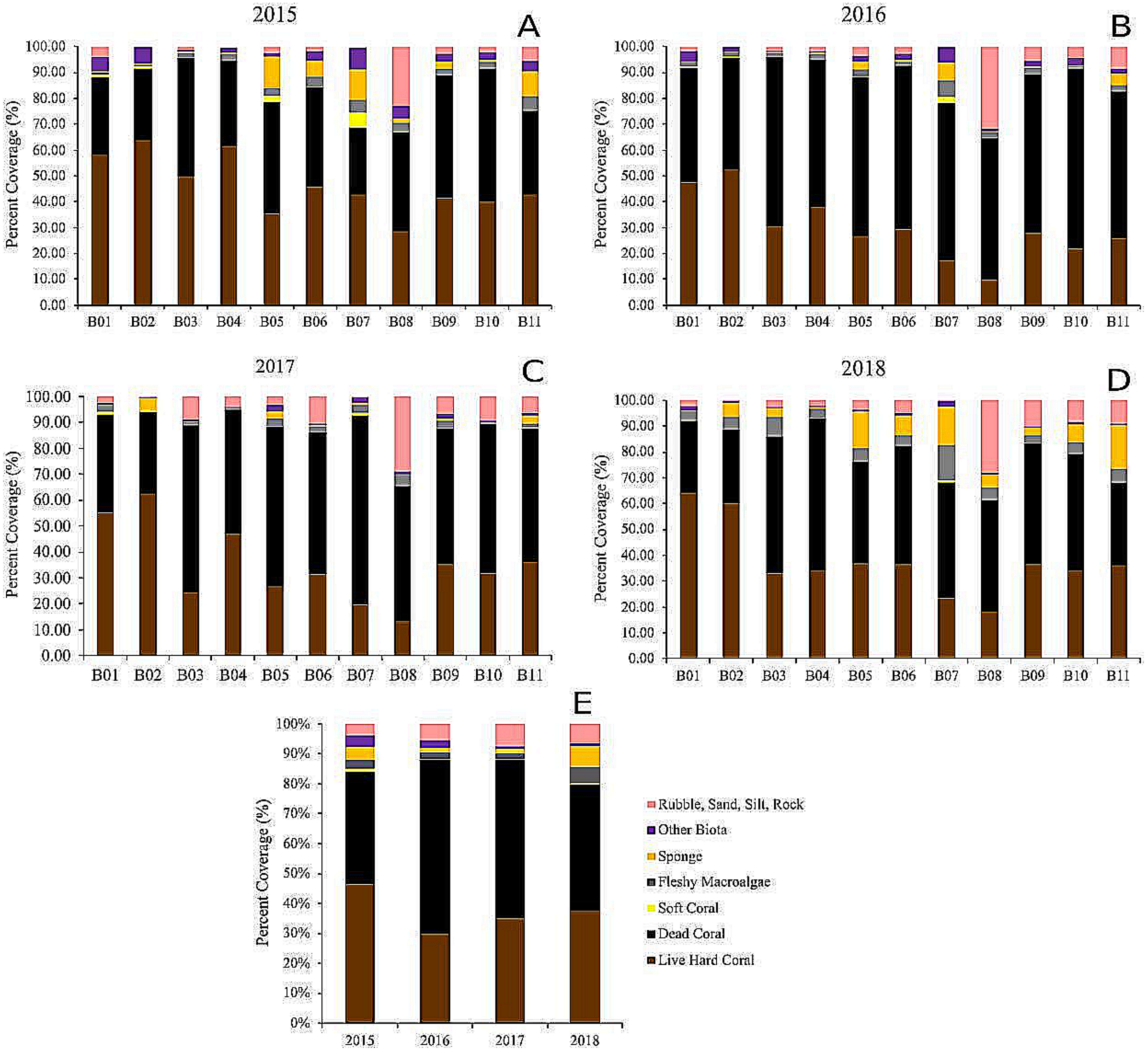
The percentage of benthic and substrate categories at each site throughout the years, 2015 (A), 2016 (B), 2017 (C), 2018 (D), and the averages (E).

**Figure 6:**
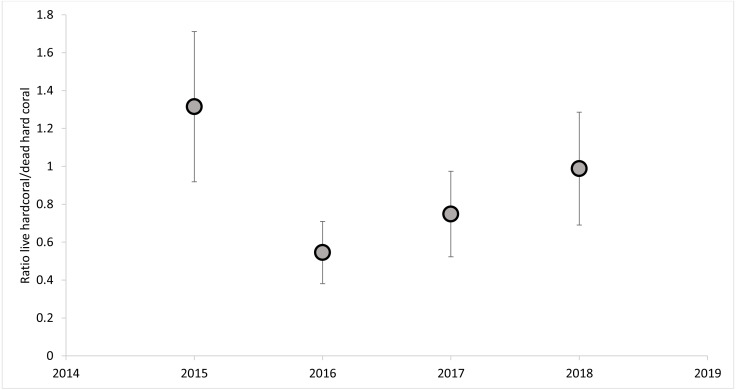
The percent cover ratios between live and dead hard corals across the years.

The dead hard coral cover category was the highest of all categories in all of the sites, revealing live *vs.* dead hard coral cover patterns (Fig. 5). The live hard coral cover across the sites and years averaged 37% and ranged from 30% to 46% cover. Dead hard coral averaged 49% and ranged from 38% to 58% cover across sites and years (Fig. 5). From 2015 to 2018, it was observed that the ratio of live hard coral *vs.* dead hard coral is as follows: 1.31, 0.46, 0.71, and 1.11 (Fig. 6). This ratio between live and dead hard corals shows the coral cover increased after the decline in 2016, signaling that the corals are in the recovery process. Soft coral cover averaged less than 0.5%, ranging from 0.1% to 0.9% through sites and years. Live hard coral cover averaged 38% cover across the sites and years and ranged from 30% to 47% (Fig. 5). As found in soft coral, the other components, *i.e.,* fleshy macroalgae, sponge, other biotas, and rubble, sand, silt, and rock, showed insignificant covers with less than 6% cover across years (Fig. 5).

The highest cover for live hard coral was found at B01 in 2018 with 64%, and the minimum value was at B08 in 2016 with a 10% cover. For dead hard coral, the highest cover was at B07 in 2017 with 73%, and the lowest was 26% at the same site in 2015. The soft coral cover reached the highest at B07 in 2015 at 6%, and the soft coral cover was null at four sites in 2015, six sites in 2016, five sites in 2017, and nine sites in 2018 (Fig. 5). The highest cover for live hard coral was at B02 in 2015 with 65%, and the lowest was at B08 in 2016 with a 10% cover (Fig. 5). The maximum value for rubble, sand, silt, and rock was at B08 in 2016 with 31%, and there was no cover at B02 in 2015. The highest sponge and other biotas value was less than 15% (Fig. 5). There was an increasing mean cover of fleshy macroalgae across the sites from 2015 to 2018. The highest cover for fleshy macroalgae was at B11 in 2018 with 16.5%, and the cover of fleshy macroalgae was null at one site (B10) in 2016 and three sites (B01, B04, and B08) in 2017 (Fig. 5).

**Figure 7:**
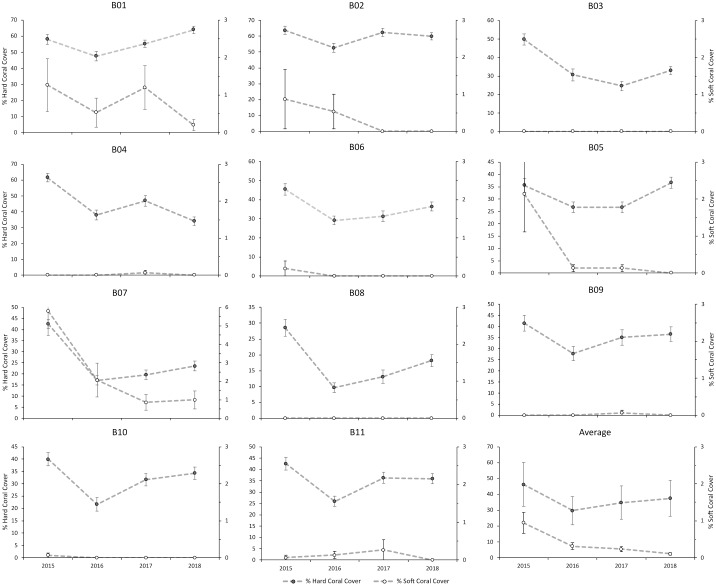
The hard and soft corals’ percent covers at each site from 2015 to 2018.

There was a slightly increasing trend in hard coral cover at all of the sites, except at B04, which decreased by 27.4% over three years (Fig. 7). Repeated measures ANOVA showed significant changes in hard coral cover from 2015 to 2018 (*P* < 0.05) (Table S1). The increase in coral cover is the result of the dominance of a few species from the family Poritidae and Faviidae (Fig. 7). In contrast, there was a downward trend in soft coral cover for all of the sites from 2015 to 2018. The most significant decrease in the cover of soft coral was in site B07, which decreased by 4.8% over three years (Fig. S1). Repeated measures ANOVA showed significant changes in soft coral cover from 2015 to 2018 (*P* < 0.05) (Table S2).

The percent cover of the five most dominant hard coral families varied across all of the sites (Fig. S1). These were the five most dominant families in all sites: Acroporidae, Faviidae, Oculinidae, Pectiniidae, and Poritidae, with the most dominant genera, were *Montipora*, *Echinopora*, *Galaxea*, *Merulina*, and *Porites*, respectively. Poritidae were the coral family with the highest cover among others, with Porites as the most contributing genera (8.7% over 9.2% on average) compared to other genera in this family (*Goniopora* and *Alveopora*). The cover of these dominant families differed among taxa (*P* < 0.05) but did not vary over the years (*P* > 0.05) (Table S3). Oculinidae were less common across all sites, except for B02, where its coverage was more than 40% for all years (Fig. S1). The percentage cover of Pectinidae was more than 5% in most of the sites, but there was zero percentage at B02 and B07 from 2016 to 2018 (Fig. S1). The percentage cover of Acroporidae was below 10% in all of the sites except at B04. Acroporidae and Poritidae were two families found in all sites throughout the years (Fig. S1). The highest coverage for both families was nearly 40%, at B01 in 2018 for Poritidae and B04 in 2017 for Acroporidae (Fig. S1). Faviidae had the highest coverage of more than 15% in two sites, B06 and B09, for all of the years (Fig. S1). The average percentage cover across the sites and years of all hard coral individual species was less than 10%. The highest percentage was only 9.6% for *Galaxea astreata* (Table 2).

## Discussion

We found a moderate richness of hard coral species across the sites and years (Table S4), although the species richness declined by 9.95% in 2016 (Fig. 2). The moderate species richness of hard corals is closely related to the distance from the biodiversity hotspot located in the eastern part of Indonesia (Asaad et al., 2018). However, the species richness in Bangka Belitung Islands is approximately two times higher compared to the western part of Indonesia, especially on the west coast of Sumatra Island (Siringoringo et al., 2017; Siringoringo et al., 2018; Utama & Hadi, 2018). The decline in species richness was primarily associated with a reduction in hard coral cover in 2016, as previously confirmed by other studies (Ampou et al., 2017; Madin et al., 2018). Nevertheless, the decline in Bangka Belitung Islands did not change the coral composition, particularly at the genera level. Furthermore, the composition was relatively similar during the monitoring period but different among the sites, making spatial variation more influential than temporal variation.

This study’s live-dead hard coral patterns highlighted the urgent need to conserve areas with high coral covers. These areas might play an essential role as refugia and as a source of larval supply for degraded sites (Edmunds, 2002). The hard corals surveyed in this biased selection of best study sites were relatively good, with high cover and a slightly increasing trend at all sites across years (except at B04, Fig. 5). These results match reports of high coral cover in tropical areas (Porter, 1974; Salvat, 2002) and Seribu Islands, Indonesia (De Vantier et al., 1995).

Even though this study used the dead coral category, which included recently dead corals as well as long-dead colonies from previous disturbances, the high percentage of dead coral cover was more likely due to rising sea surface temperature, especially around April to July, as reported by NOAA coral reef watch (Kimura, Tun & Chou, 2018). Other studies in Bunaken and Lombok have also reported that coral coverage declined due to heat stress in 2016 (Ampou et al., 2017; Bachtiar & Hadi, 2019; McClanahan et al., 2019). According to the NOAA, coral reef watch warning, bleaching warning, and alert one status have been reported at Bangka Belitung with temperatures over 29 °C between May and August 2016 (Fig. S2). The inability of coral reefs to recover after multiple bleaching events in a row due to prolonged stresses, such as changes in light and temperature, pollution, disease, and freshwater flooding (Douglas, 2003), might exacerbate coral reef conditions. Moreover, the environment might not stabilize enough so that corals could not regrow their zooxanthellae and recover from disturbance (Douglas, 2003).

We found a slightly increasing trend in hard coral cover after the decline in 2016, and repeated measures of ANOVA confirmed that there were changes during the period (*P* < 0.05), suggesting that these reef corals are among the relatively high cover reef in the world. However, the hard coral cover is a rough representation of disturbance (Connell, 1978). This finding contrasts with Van Der Meij, Suharsono & Hoeksema (2010), who stated the coral reef decline patterns, nevertheless supporting the GCRMN (Global Coral Reef Monitoring Network) report (Souter et al., 2021), which reported reef increasing patterns in Indonesia. The increasing trend after the 2016 heat stress might be due to the hard corals’ recovery process, mainly associated with the coral composition. Apart from the fast-growing coral composition, we found that faviid corals were able to increase their number of species, and thus, these species contributed to the coral cover increase, while the poritiid corals contributed largely to the increase of coral cover as two species, especially *Porites lobata* and *P. Lutea*, were among the most dominant species found in this study. These two species are predicted to dominate Bangka Belitung waters in the future if the sea surface temperature in the area gets warmer, as found in other reefs (Yu et al., 2019).

Fleshy macroalgae have been used as an indicator of nutrient enrichment due to sewage pollution (Hodgson, 1999). Our study revealed a low cover of fleshy macroalgae that was possibly due to the choice of sites located on the outer slopes of the reefs, which were relatively far from urban areas. We also did not find that the sites with the lowest fleshy macroalgae covers coincided with the most excellent live hard coral cover site, as found in others research (Evans et al., 2020). The five most dominant families we found with the highest mean cover across sites and years are essential in driving coral dynamics (Osborne et al., 2011; Wilson et al., 2019). Corals from these families are typically competitive and fast-growing taxa known as the main driver of recovery on other reefs (Darling et al., 2012; Johns, Osborne & Logan, 2014; Doropoulos et al., 2015; Wilson et al., 2019). Sponge and other biotas only have a limited cover (<15%), and this low percentage cover of sponges has also been reported previously in the Indo-Pacific (Hodgson, 1999). *Galaxea astreata* was the most abundant coral species as it has the highest average percentage cover (9.6%) and occurred in eight out of 11 sites across sites and years. Similarly, Cheng & Dai (2018) reported that *Galaxea astreata* is a widely distributed scleractinian coral in the South China Sea, even though *Galaxea* is a small Indo-Pacific genus with only nine accepted species (Hoeksema & Cairns, 2021). An experimental study using flow chambers on the ability of hard coral species to prey on coral larvae reported that *Galaxea astreata* was an effective predator, which might affect reef recruitment rates (Fabricius & Metzner, 2004).

The three genera of hard corals, *i.e., Acropora*, *Montipora*, *Porites*, that we found with the most species members are species-rich coral genera occurring on most tropical reefs and were reported to have remarkable diversity in Indonesia (Wallace, 1999; Wallace, 2001; Van Oppen, 2004; Xu et al., 2017). *Acropora* and *Montipora* corals are known to have fast growth rates (Kayanne et al., 2002; Van Oppen, 2004; Anderson, Pratchett & Baird, 2012), and *Porites* corals are the most resistant genus to thermal stress (Edwards et al., 2001; Stimson, Sakai & Sembali, 2002; Xu et al., 2017). A combination of these types of corals largely contributes to reef resilience, especially in dealing with the bleaching event and the recovery process afterward. In addition, nine hard coral species (*i.e., Porites lutea*, *Physogyra lichtensteini*, *Galaxea fascicularis*, *Fungia concinna*, *Merulina scabricula*, *Porites cylindrica*, *Fungia* sp., *Porites* sp., and *Galaxea* sp.) in all study sites were relatively common in Indonesian waters (Suharsono, 2008). This finding indicates that the reefs might be connected to each other in terms of larval dispersal, or the corals are categorized as old species (Hoeksema, 2007).

## Conclusions

The coral cover in Bangka Belitung Islands is very dynamic in response to the change in environmental conditions, particularly the sea surface temperature, but it does not change the hard coral community structure at the genera level. The community structure plays an important role in the recovery process after disturbance. It is advised that Bangka Belitung reefs of good coral diversity and a high potential for recovery are essential to study and protect because they can be baselines for restoration projects and valuable sources of coral larvae or donor colonies for coral propagation. This study suggests that the hard coral community structure and richness positively impact the recovery process after the heat stress event. Identifying areas that are recovering or stable despite apparent anthropogenic and natural variations is critical to guiding management policies in the face of climate change.

##  Supplemental Information

10.7717/peerj.14625/supp-1 Supplemental Information 1 Percentage cover (±SE) and species richness of the dominant families of hard coral across the 11 study sites in nine islands (Batu Malang, Penyu, Lengkuas, Mendanau, Batudinding, Sekutai, Sebongkok, Naduk, Sepindang, and Ruk) in The Bangka Belitung provi

10.7717/peerj.14625/supp-2 Supplemental Information 2 Sea surface temperature patterns and degree heating weeks (DHW) in Belitung waters between 2015-2018 (NOAA Reef Watch, 2022)

10.7717/peerj.14625/supp-3 Supplemental Information 3 Summary of the ANOVA test to analyze changes in hard coral cover from 2015 to 2018. df: degrees of freedom

10.7717/peerj.14625/supp-4 Supplemental Information 4 Summary of the ANOVA test to analyze changes in soft coral cover from 2015 to 2018. df: degrees of freedom

10.7717/peerj.14625/supp-5 Supplemental Information 5 Summary of the ANOVA test to analyze the cover of five dominant families among taxa, and over the years from 2015 to 2018. df: degrees of freedom

10.7717/peerj.14625/supp-6 Supplemental Information 6 Comparison of the number of hard coral species and live coral covers using the photo quadrant method in Indonesia

## Additional Information and Declarations

### Data Availability

The following information was supplied regarding data availability: The raw data is available in the Supplemental Files.
