# Supplementary figures and images for: Species richness and the dynamics of coral cover in Bangka Belitung Islands, Indonesia
Source: PeerJ. 2023 Feb 24;11:e14625. doi: 10.7717/peerj.14625 (PMC9969856; doi:10.7717/peerj.14625)

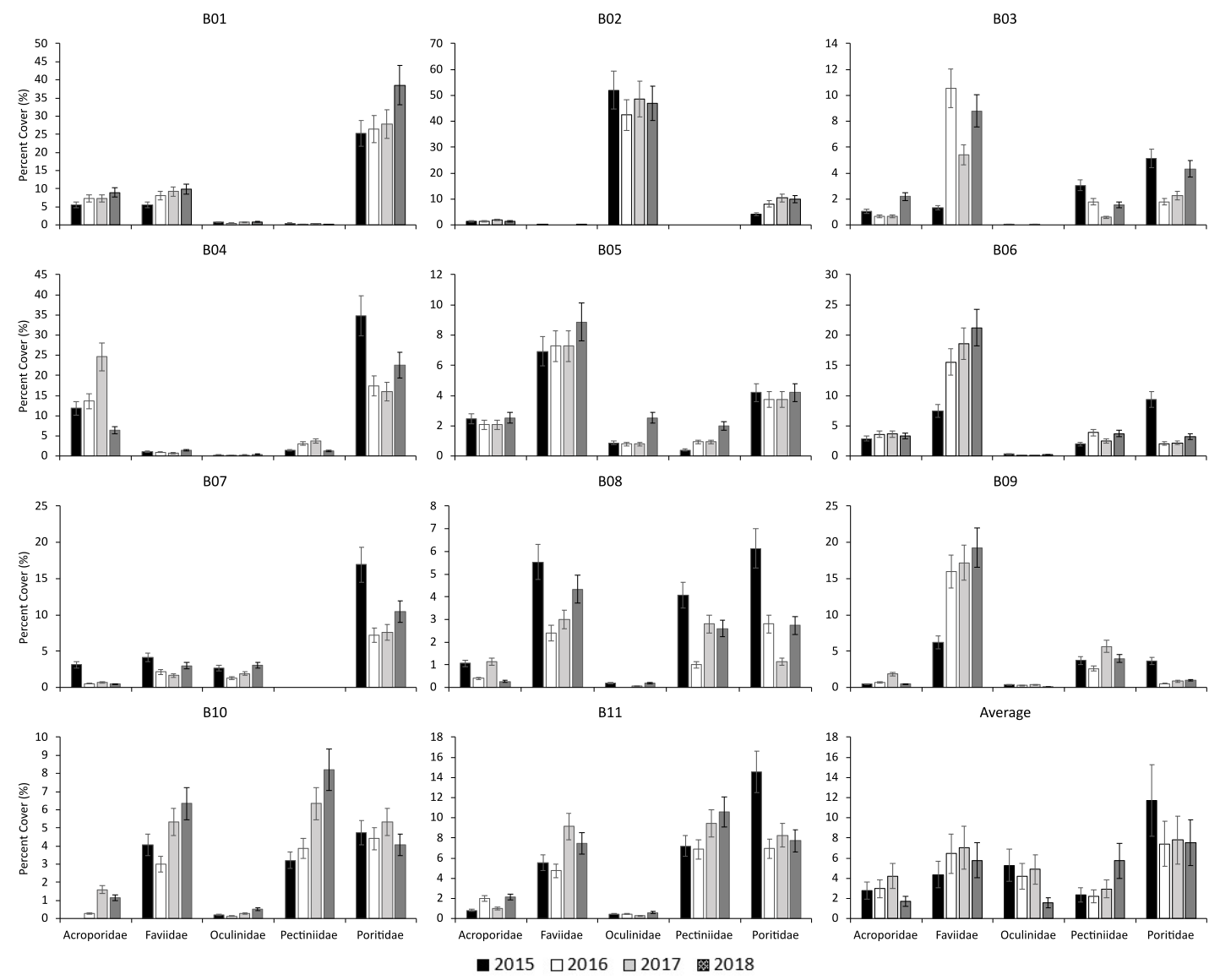

Supplement: Supplemental Information 1 [file peerj-11-14625-s001.png]

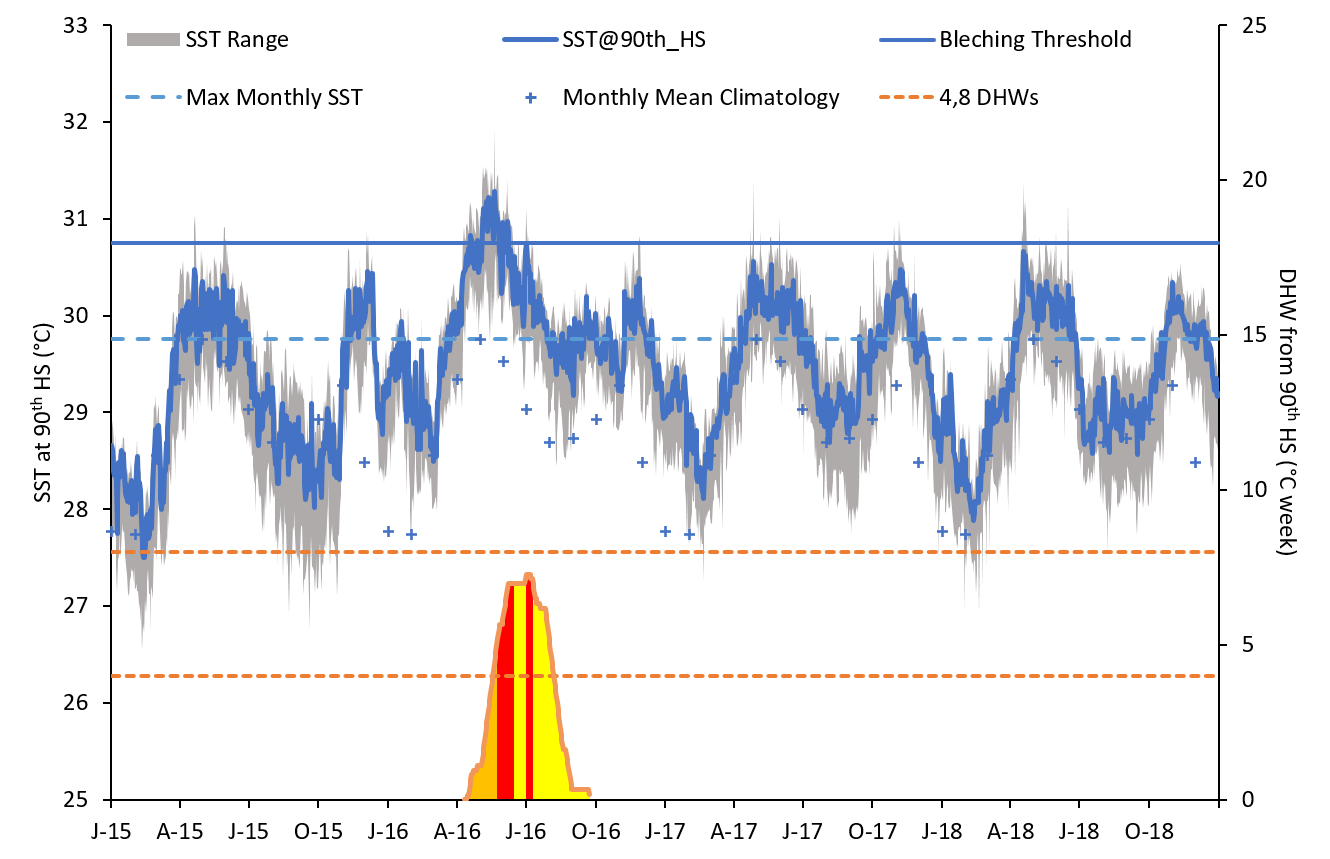

Supplement: Supplemental Information 2 [file peerj-11-14625-s002.png]
