# Supplementary material for: Species richness and the dynamics of coral cover in Bangka Belitung Islands, Indonesia
Source: PeerJ. 2023 Feb 24;11:e14625. doi: 10.7717/peerj.14625 (PMC9969856; doi:10.7717/peerj.14625)

**Table S1.** Summary of the ANOVA test to analyze changes in hard coral cover from 2015 to 2018. df: degrees of freedom.


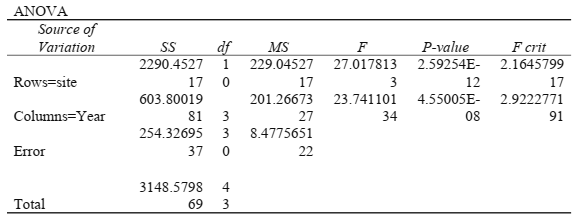

Supplement: Supplemental Information 3 [file peerj-11-14625-s003.docx]
