# Supplementary material for: Species richness and the dynamics of coral cover in Bangka Belitung Islands, Indonesia
Source: PeerJ. 2023 Feb 24;11:e14625. doi: 10.7717/peerj.14625 (PMC9969856; doi:10.7717/peerj.14625)

**Table S2.** Summary of the ANOVA test to analyze changes in soft coral cover from 2015 to 2018. df: degrees of freedom


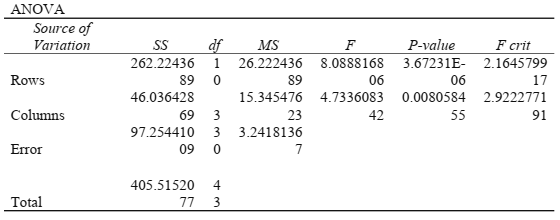

Supplement: Supplemental Information 4 [file peerj-11-14625-s004.docx]
