# Supplementary material for: Species richness and the dynamics of coral cover in Bangka Belitung Islands, Indonesia
Source: PeerJ. 2023 Feb 24;11:e14625. doi: 10.7717/peerj.14625 (PMC9969856; doi:10.7717/peerj.14625)

**Table S3.** Summary of the ANOVA test to analyze the cover of five dominant families among taxa, and over the years from 2015 to 2018. df: degrees of freedom


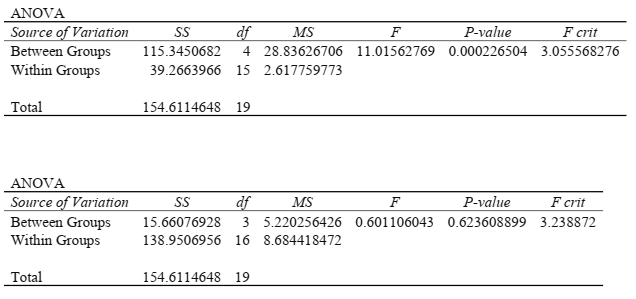

Supplement: Supplemental Information 5 [file peerj-11-14625-s005.docx]
