# Supplementary material for: Species richness and the dynamics of coral cover in Bangka Belitung Islands, Indonesia
Source: PeerJ. 2023 Feb 24;11:e14625. doi: 10.7717/peerj.14625 (PMC9969856; doi:10.7717/peerj.14625)
Supplement: Supplemental Information 6 [file peerj-11-14625-s006.docx]

**Supplement Table S4.** Comparison of the number of hard coral species and live coral covers using the photo quadrant method in Indonesia.

| Number of study sites | Time  (in year) | Total species richness in the study area | Average species richness per site | Genera Richness | Average Live Coral Cover (%) | Reference |
| --- | --- | --- | --- | --- | --- | --- |
| 13 | 2020 | 398 | 132 | 68 | 28.46 | Dharmawan et al. 2020 |
| 12 | 2019 | 394 | 153 | 72 | 24.62 | Suyarso et al. 2019 |
| 11 | 2018 | 249 | 59 | 52 | 37.50 | Current study |
| 11 | 2015 | 208 | 58 | 53 | 46.29 | Current study |
| 11 | 2016 | 189 | 46 | 49 | 29.72 | Current study |
| 11 | 2017 | 177 | 23 | 52 | 34.82 | Current study |
| 13 | 2018 | 158 | Not mentioned | Not mentioned | 24.77 | Siringoringo et al. 2018 |
| 10 | 2017 | 137 | Not mentioned | Not mentioned | 11.33 | Siringoringo et al. 2017 |
| 5 | 2016 | 133 | Not mentioned | Not mentioned | 29.97 | Hadi et al. 2019 |
| 5 | 2014 | 130 | Not mentioned | Not mentioned | 33.58 | Hadi et al. 2019 |
| 10 | 2017 | 82 | 20 | 30 | 28.48 | Utama and Hadi 2018 |
